# Joint optic disc and cup segmentation based on densely connected depthwise separable convolution deep network

**DOI:** 10.1186/s12880-020-00528-6

**Published:** 2021-01-28

**Authors:** Bingyan Liu, Daru Pan, Hui Song

**Affiliations:** South China Normal University, Guangzhou, 510006 China

**Keywords:** Deep learining, Optic disc segmentation, Optic cup segmentation, Depthwise separable convolution, Densely connected

## Abstract

**Background:**

Glaucoma is an eye disease that causes vision loss and even blindness. The cup to disc ratio (CDR) is an important indicator for glaucoma screening and diagnosis. Accurate segmentation for the optic disc and cup helps obtain CDR. Although many deep learning-based methods have been proposed to segment the disc and cup for fundus image, achieving highly accurate segmentation performance is still a great challenge due to the heavy overlap between the optic disc and cup.

**Methods:**

In this paper, we propose a two-stage method where the optic disc is firstly located and then the optic disc and cup are segmented jointly according to the interesting areas. Also, we consider the joint optic disc and cup segmentation task as a multi-category semantic segmentation task for which a deep learning-based model named DDSC-Net (densely connected depthwise separable convolution network) is proposed. Specifically, we employ depthwise separable convolutional layer and image pyramid input to form a deeper and wider network to improve segmentation performance. Finally, we evaluate our method on two publicly available datasets, Drishti-GS and REFUGE dataset.

**Results:**

The experiment results show that the proposed method outperforms state-of-the-art methods, such as pOSAL, GL-Net, M-Net and Stack-U-Net in terms of disc coefficients, with the scores of 0.9780 (optic disc) and 0.9123 (optic cup) on the DRISHTI-GS dataset, and the scores of 0.9601 (optic disc) and 0.8903 (optic cup) on the REFUGE dataset. Particularly, in the more challenging optic cup segmentation task, our method outperforms GL-Net by 0.7$\%$ in terms of disc coefficients on the Drishti-GS dataset and outperforms pOSAL by 0.79$\%$ on the REFUGE dataset, respectively.

**Conclusions:**

The promising segmentation performances reveal that our method has the potential in assisting the screening and diagnosis of glaucoma.

## Background

Glaucoma is an eye disease that damages the optic nerves and causes irreversible vision loss [1]. It has been estimated that 60.5 million people globally were affected by glaucoma in 2010 and predicted to affect almost 80 million people worldwide by 2020 [2]. Since vision loss is irreversible, early detection and diagnosis are very important to prevent vision loss and has been shown to decrease the rate of blindness by around 50$\%$ [3]. Hence it is essential to have a glaucoma screening technique to identify glaucomatous and healthy eyes. Intraocular pressure assessment (IOP), visual field test and optic nerve head (ONH) assessment are three main techniques to detect glaucoma, in which ONH evaluation is the most clinically significant screening technique for glaucoma. For ONH evaluation, cup to disc ratio (CDR), means optic nerve rim to disc ratio in diameters, is one of the most important indicators for glaucoma screening and diagnosis. Accurate segmentation of optic disc (OD) and optic cup (OC) is essential for the calculation of CDR. However, manual calculation of CDR by experienced clinicians is time-consuming and expensive and is not suitable for population screening for glaucoma. Therefore, computer-aided diagnosis (CAD) methods for large-scale fundus image screening are needed. Segmenting the optic disc and optic cup is the preliminary step in CDR measurement and Glaucoma assessment. Many works have been proposed to segment the optic disc and cup from the fundus image to assist clinicians to diagnose glaucoma more effectively.

**Fig. 1:**
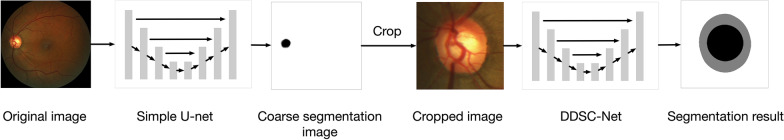
Overview of our proposed method. Firstly, simple DDSC-Net without multi input is used to rough segment the optic disc, and located the optic disc by CHT. Then fed cropped image into segmentation network to joint segment OD and OC

**Fig. 2:**
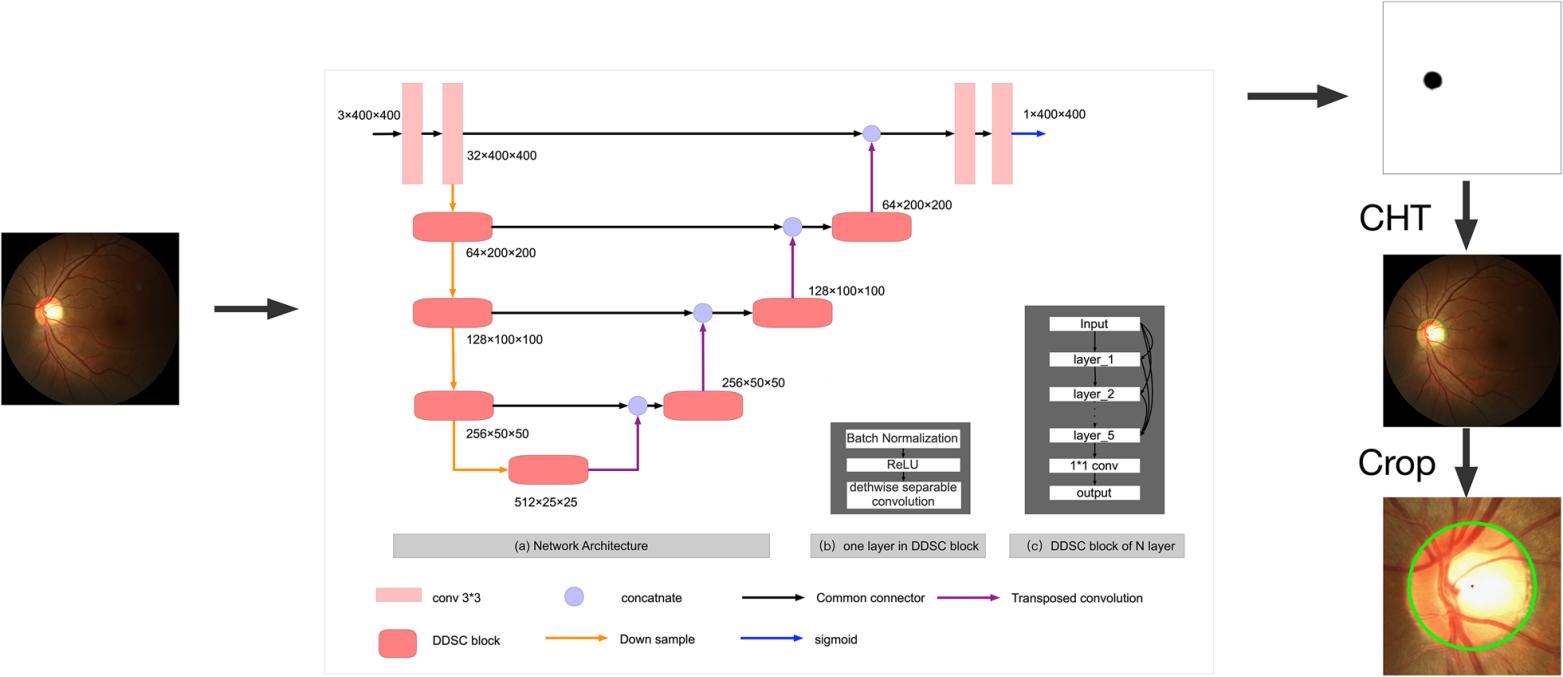
ROI extraction model. Simple DDSC-Net architecture is a simple version of DDSC-Net. After obtain the OD segmentation result, CHT is employed to calculate the center and radius of OD.ROI is a small image cut out from the center of the calculated circle

**Fig. 3:**
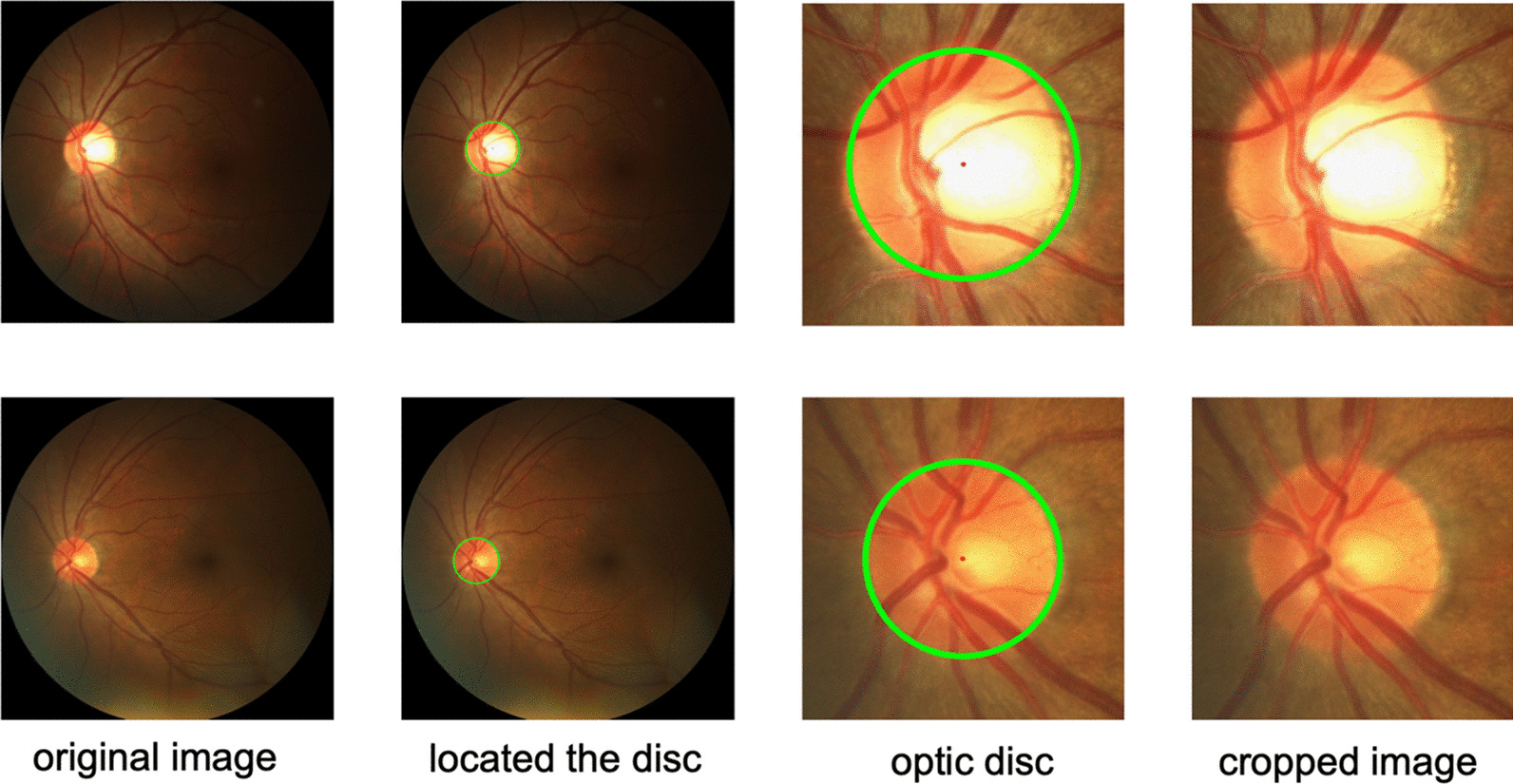
Location and cropping results of ROI. The first column of images is the original images, the second column is the position of the optic disc located, the third and fourth is the position of the optic disc after cropping and the cropping result

**Fig. 4:**
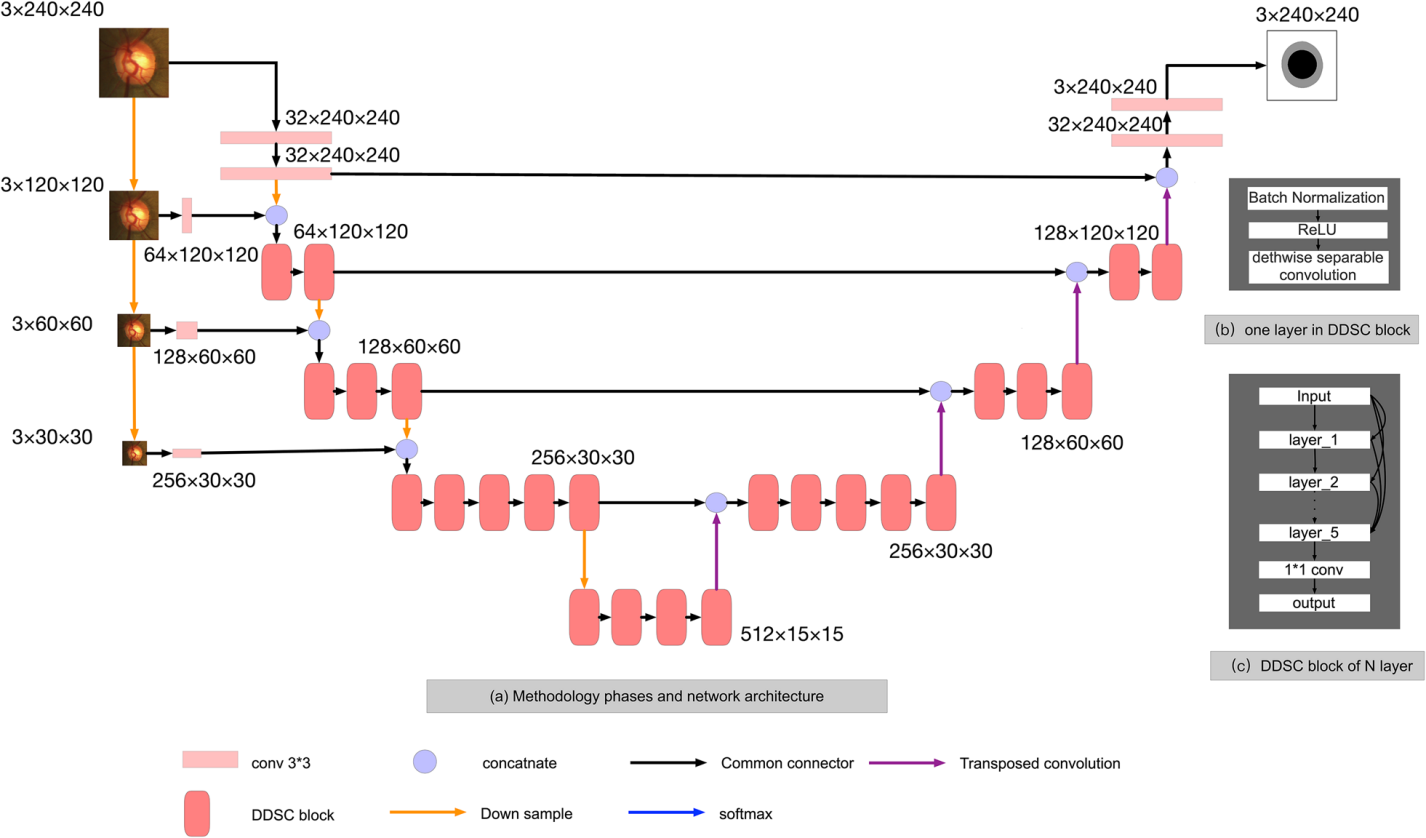
Network architecture of the DDSC-Net. Our DDSC-Net architecture includes multi-scale input of image pyramid and DDSC-blocks. As shown in the figure, each DDSC-block is composed of five “BN + Relu + depthwise separable convolution” dense connection layers, the convolution kernel is 3 x 3.The down sample is the maximum pooling layer, and the upsample is transposed convolution. Finally, the output of the network uses a softmax activation function to classify the output map into three categories: 0, 1 and 2. 0 is the background, category 1 is the optic disc, and 2 is the optic cup

**Fig. 5:**
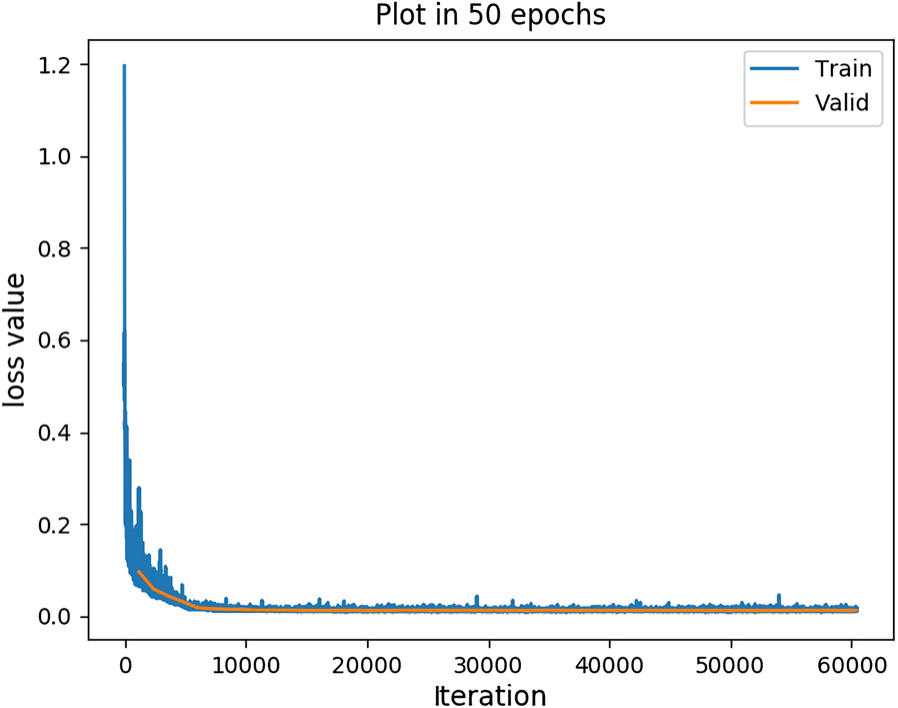
Training and validation loss on the DRISHTI-GS dataset. The blue line is the training loss, and the orange line is the verification loss

**Fig. 6:**
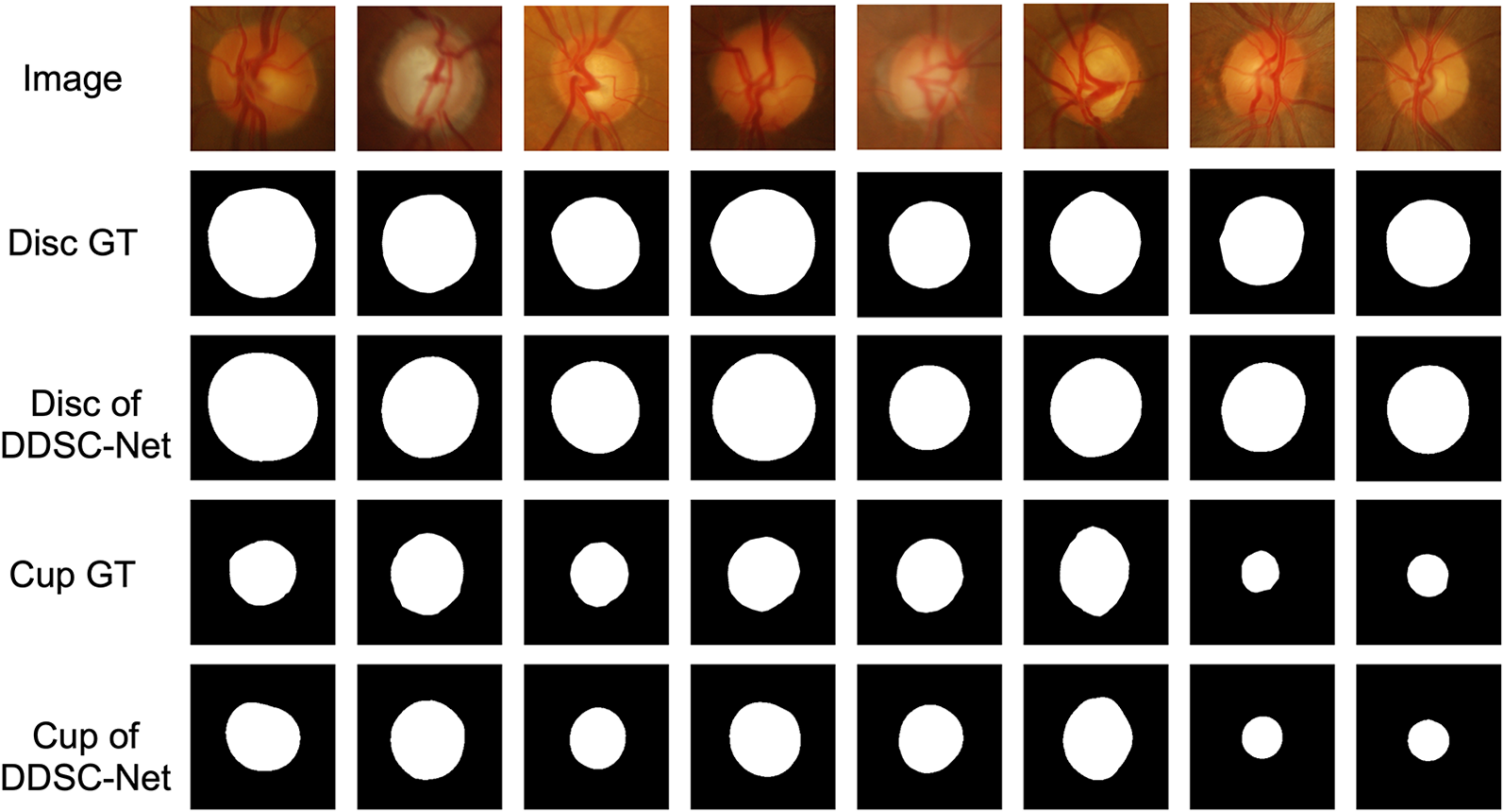
The visual examples of OD and OC segmentation on DRISHTI-GS dataset. The first row is the input image, the second and fourth rows are the ground truth of the OD and the OC respectively, and the third and fifth rows are the OD, OC segmentation results of our model, where the black denotes the background, and the white part denotes the OD and OC segmentations

**Fig. 7:**
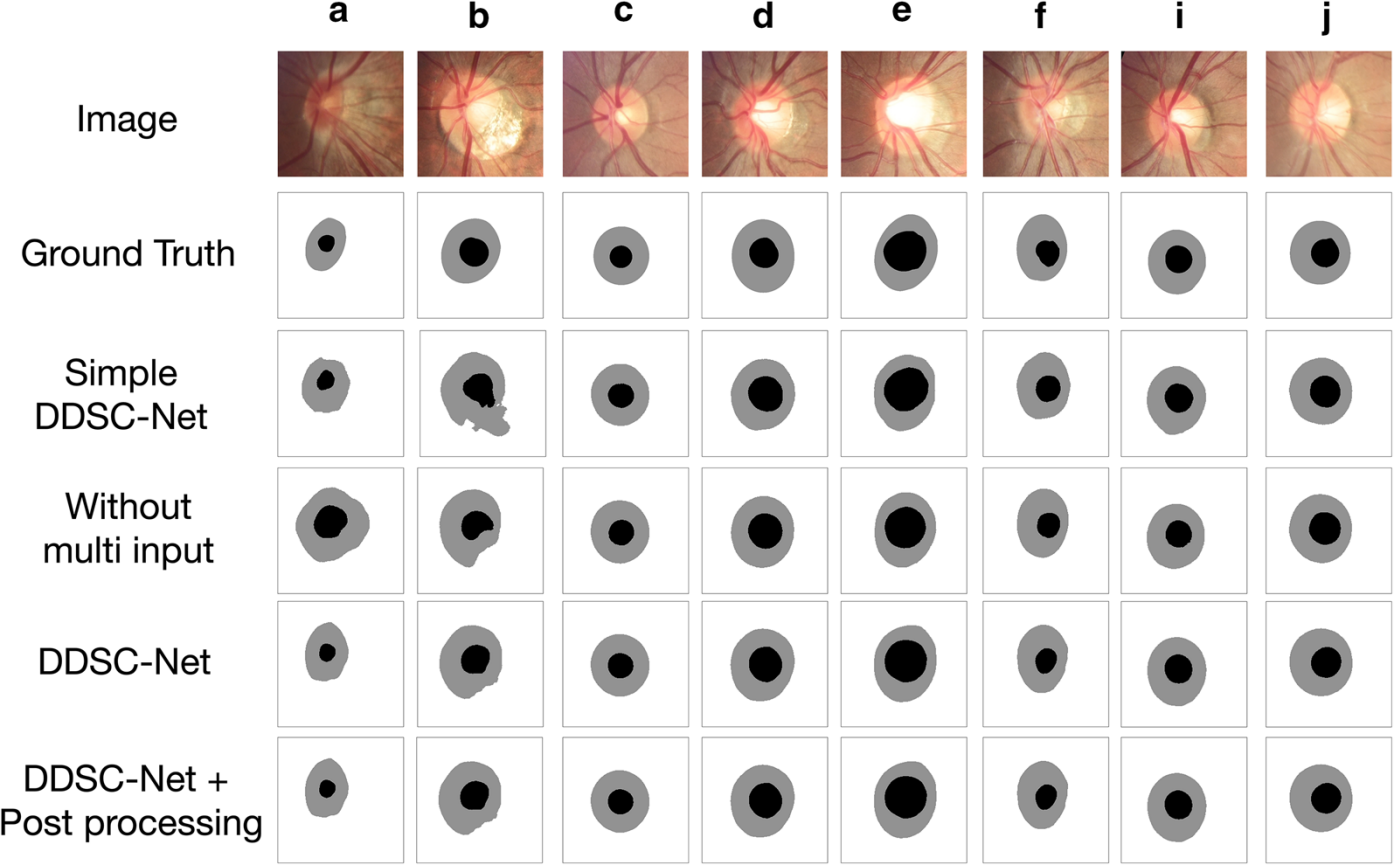
Some ablation experiment results on REFUGE dataset. The first row is the input image, the second is the ground truth, the third row is the segmentation result of simple DDSC-Net, the fourth row is the result of the DDSC-Net without image pyramid input, the fifth row is the result of DDSC-Net and the sixth row is the reprocess result, where the white region denotes the background, and the gray and black region denotes the OD and OC segmentations

The main segmentation techniques include template-based methods [4, 5], boundary detection [6, 7], hand-crafted visual feature approach, and deep learning segmentation methods [8–11]. In these methods, the template-based method models the OD as s circular or elliptical object and employed a circular Hough transforms [4, 5] or sliding band filter [6] to obtain an approximate boundary of the optic disc. The method based on boundary detection needs to mark multiple landmark points [6], or requires each pixel to have a direct edge in the 15-pixel neighborhood, and does not consider depth information [7]. The hand-crafted visual feature approaches convert boundary problems into pixel classification problems, and obtains satisfactory results. However, due to the quality change of the fundus image and the presence of internal and surrounding blood vessels, the precise boundary of the optic disc and cup cannot be robustly obtained through these approaches.

**Table 1:** Statistics of the dataset used in evaluation the proposed method

DATASET	Train and val	Test	Total	Image size	Release year
DRISHTI-GS	50	51	101	$2047\times 1759$	2014
REFUGE	800	400	1200	$2124\times 2056$ and $1634\times 1634$	2018

**Table 2:** Localization accuracy results

Dataset	Images accurately located	Total	Accuracy
DRISHTI-GS	101	101	1.0
REFUGE	1200	1200	1.0

**Table 3:** Experiment results

DATASET	OD				OC			
	*DC*	*JAC*	*SEN*	*PRE*	*DC*	*JAC*	*SEN*	*PRE*
DRISHTI-GS	0.9780	0.9570	0.9784	0.9778	0.9123	0.8442	0.9220	0.9149
REFUGE	0.9601	0.9239	0.9814	0.9412	0.8903	0.8065	0.9209	0.8749

**Table 4:** The model segmentation results with and without Multi input, deeper network and post-processing

Method	DRISHTI-GS dataset		REFUGE dataset	
	$DC_{disc}\,(mean)$	$DC_{cup}\,(mean)$	$DC_{disc}\,(mean)$	$DC_{cup}\,(mean)$
Without image pyramid input	0.9743	0.9076	0.9589	0.8880
Without multi-DDSC blocks	0.9746	0.8939	0.9575	0.8837
DDSC-Net	0.9779	0.9123	0.9592	0.8894
DDSC-NET + post-processing	*0.9780*	*0.9123*	*0.9601*	*0.8903*

Italics indicate the best results

**Table 5:** Comparison with the deep learning methods on DRISHITI-GS dataset

Method	$DC_{disc}\,(mean)$	$DC_{cup}\,(mean)$	$JC_{disc}\,(mean)$	$JC_{cup}\,(mean)$
U-Net [15]	0.950	0.820	–	–
M-Net [16]	0.959	0.866	–	–
Stack-U-Net [30]	0.970	0.890	–	–
Sevastopolsky [10]	–	0.850	–	0.750
Yu et al. [34]	0.973	0.887	–	–
MULTI-MODEL-PRE-TRAINING [33]	0.960	0.902	0.924	0.823
WGAN [31]	0.954	0.840	0.912	0.724
pOSAL [31]	0.965	0.858	–	–
GL-Net [29]	0.971	0.905	–	–
Proposed	*0.978*	*0.912*	*0.957*	*0.844*

Italics indicate the best results

**Table 6:** Comparison with the deep learning methods on REFUGE dataset

Method	$DC_{disc}\,(mean)$	$DC_{cup}\,(mean)$	$JC_{disc}\,(mean)$	$JC_{cup}\,(mean)$
M-Net [16]	0.9436	0.8315	–	–
MULTI-MODEL-PRE-TRAINING [33]	–	–	0.9225	0.7902
Two-stage Mask R-CNN [32]	0.9504	0.8546	–	–
pOSAL [31]	*0.9602*	0.8826	–	–
Proposed	0.9601	*0.8903*	*0.9239*	*0.8065*

Italics indicate the best results

**Table 7:** Result of OD and OC segmentation on the REFUGE dataset

TEAM	$DC_{disc}\,(mean)$	$DC_{cup}\,(mean)$
CHUKMED	*0.9602*	*0.8826*
Masker	0.9496	*0.8837*
BUCT	0.9525	0.8728
NKSG	0.9488	0.8643
VRT	*0.9532*	0.8600
AIML	0.9505	0.8519
Mammoth	0.9361	0.8667
SMILEDeepDR	0.9386	0.8367
NightOwl	0.9487	0.8257
SDSAIRC	0.9436	0.8315
Cvblab	0.9077	0.7728
WinterFell	0.8772	0.6861
Proposed	*0.9601*	*0.8903*

Top three results are marked in italics

It has been shown that deep learning-based techniques, represented by convolution neural network (CNN) [12], achieve promising results for optic disc and optic cup segmentation [8–11]. Compared with the aforementioned hand-crafted design feature extraction methods, deep learning networks based on convolutional neural networks can automatically extract complex features from the input data. In [8], an optic disc and optic cup segmentation method based on CNN which used an entropy-based sampling technique to reduce computational complexity is proposed. Although the segmentation performance of this approach is better than that of the method of using hand crafted features, it leaves much to desire in terms of time-consuming. In [9], a fully convolutional neural network [13] based on VGG-16 net [14] is proposed to segment the optic disc. This method can segment retinal vessels and optic disc simultaneously, without segmenting the more challenging OC. In 2015, Ronneberger et al. [15] proposes an architecture called U-Net, which has been widely used in biomedical image segmentation and has achieved good results. And many biological image segmentation networks are modified based on the U-Net convolutional network. In [10], a modified version of the U-Net convolutional network is presented for automatic optic disc and cup segmentation. Although this method has the advantages of fast processing speed and fewer parameters, it fails to make full of the context information of the network, which results in the poor segmentation of OC. In [11], the authors utilize fully convolutional networks with adversarial training for jointly segment the OD and OC, but assumes that the region of interest (ROI) can be accurately extracted by preprocessing. Furthermore, Fu et al. [16], proposes a deep learning architecture named M-Net which used a polar transformation with the multi-label deep learning concept. Although M-Net uses the same network architecture to segment the optic disc and the cup, it still considers these two segmentation tasks as two independent problems.

At present, most segmentation methods consider the process of detection-then-segmentation mechanism. However, many location methods are susceptible to the influence of the image quality and pathological area [8, 10], resulting in wrong location. Furthermore, the accurate segmentation of the optic cup is a more challenging task in the task of disc and cup segmentation. Although most of the disc and cup segmentation networks based on deep learning can obtain satisfactory OD segmentation results, they cannot produce more accurate optic cup segmentation results [8–11, 16].

For the above problems, the purpose of this study is to explore a novel CAD model for joint OD and OC segmentation that can assist clinicians in large-scale glaucoma screening. The aims of this paper are as follow: (1) First, we aim at exploring an OD location method based on deep learning to solve the problem of instability location of traditional hand-crafted design feature methods due to the change of fundus image quality and the influence of pathological areas; (2) Second, we aim at exploring a deeper and wider CNN model structure to obtain richer and more complex fine-grained features in fundus images, so that the model can perform better OD and OC segmentation results, especially in the more difficult OC segmentation task. With correct OD and OC segmentation results, accurate CDR values can be calculated to assist clinicians in screening and diagnosis.

## Methods

The method proposed in this paper employed a two-stage approach to implementing the segmentation of the optic disc and cup. In the first stage, CNN and Hough circle detection are used to obtain the center coordinates of the optic disc and extract the ROI. In the second stage, ROI is fed into the model to train a high-precision segmentation network to obtain the accurate segmentation results of optic disc and cup. The proposed method is trained and evaluated on DRISHTI-GS [17] and REFUGE datasets [18], respectively. The overall flowchart of our proposed method is shown in Fig. 1. The details of the datasets and the framework are explained in the following subsections.

### Dataset

DRISHTI-GS dataset contains 101 retinal fundus images that were collected at Aravind eye hospital, Madurai. The resolution of these images is $2047\times 1759$ and store in uncompressed PNG format. And the ground truth of these images was marked by 4 ophthalmologists with different clinical experience and divided into 50 training and 51 testing images. Retinal Fundus Glaucoma Challenge (REFUGE) dataset contains 1200 images which include 120 glaucomatous and 1080 non-glaucoma images. The REFUGE dataset is divided into three parts: 400 training images, 400 validation images and 400 testing images, in which the validation and testing images are acquired with the same cameras. Brief information about these two datasets is shown in Table 1.

In our proposed method, 50 training images on the DRISHTI-GS dataset are adopted for the training the proposed model, and the other 51 testing images are used for evaluating the performance of the final trained model. Similarly, 800 images from training set and validation set on the REFUGE dataset are utilized for training, and the other 400 images from the testing set are employed to evaluate the performance of the final model trained with the REFUGE dataset.

### Image processing and data augmentation

Because the dataset used for training has fewer images, for example, there are only 50 training images on the DRISHTI-GS dataset, and too few data for network training may lead to overfitting, so we utilize data augmentation to expand training images to prevent this problem. The augmentation methods include translation, rotation, noise addition, and brightness adjustment. Among them, the images used for training in the DRISHTI-GS dataset are expanded to 5250, and training images in the REFUGE dataset are expanded to 30,000. Specifically, 90$\%$ of the data-augmented training images are randomly selected to train the proposed model, and the rest 10$\%$ images are employed for model evaluation when training the model. For example, when using the GS dataset to train the segmentation network, 4725 images out of the 5250 images are adopted to train the segmentation network, and another 525 images are used to evaluate the model during the training process.

### ROI extraction network

Since the resolution of a complete fundus image taken by professional camera is generally relatively large, and the area of interest is only a small area in fundus image, locating and cropping out the region of interest can reduce the interference of unnecessary background information on the segmentation result, and can improve the segmentation accuracy and reduce the amount of calculation. However, the methods which employ green channel images [8] or morphological operations [19] to detect optic disc is susceptible to the effects of images taken by different devices, fundus image quality, brightness, internal blood vessels, and lesions in fundus images, resulting in low location accuracy. In our work, we utilize a method based on CNN network to extract features to solve this problem. The model and segmentation process are shown in the Fig. 2. At this stage, we design a simple convolutional neural network to segment the optic disc simply, then use Circular Hough Transform (CHT) [20] to calculate the center of the optic disc. With this method, we can locate the optic disc with 100$\%$ accuracy and crop the ROI area. The location result is shown in Table 2.

CHT is an extension of the Hough transform [21], which is mainly used to detect the circle object in the image. For circle detection, the HT is based on the equation of circle, defined as:

$$\begin{aligned} \left( x-a\right) ^{2}+\left( y-b\right) ^{2}=r^{2} \end{aligned}$$

where (*a*, *b*) represents the coordinates of circle center and *r* is radius. Center coordinates can be obtained by performing the CHT on the image. CHT can be defined as:

$$\begin{aligned} \left( P_{c}, r\right) =C H T\left( I, r_{\min }, r_{\max }\right) \end{aligned}$$

where $p_c=(i_c,j_c)$ and *r* represents the center position and the radius respectively which define the circle with the highest punctuation in the Circular Hough Transform implemented by CHT. *I* is the input image. The radius r is restricted to be between $r_{\min }$ and $r_{\max }$. In our method we set $r_{\min }$ and $r_{\max }$ as 40, 160 respectively. After obtaining the coordinates of the center of the disc, we use it as the center point to cut the original image into a small picture with a resolution of $480\times 480$ on REFUGE dataset and $560\times 560$ on DRISHTI-GS dataset. The image contains the optic disc, optic cup and some background information. The visual result examples of ROI extraction are shown in Fig. 3

### DDSC network architecture

In the object detection network [22–24], features extracted from shallow network can be used to detect small objects, while features extracted from deep network can be used to detect large objects. In the segmentation task of optic disc and optic cup, these ideas were adopted to design our network structure. Considering the prior knowledge that the optic cup is located in the optic disk, we use dense and skip connection to make full use of the context semantics of the shallow layers and deep layers. The proposed network structure is detachedly shown in the Fig. 4. The proposed deep network, named DDSC-Net, is consists of three main parts. The first part is the image pyramid [25], which is used as the multi-scale input of the network so that the network can receive image information of different scales. Multi-scale input can solve the problem of losing part of the image information with the depth of the network. The second part of our DDSC-net is a U-shaped fully convolutional network which includes an encoder module on the left and decoder module on the right. The output map is activated by the softmax activation function, and then the cross-entropy loss function is introduced to calculate the difference between the segmentation result and the real ground truth.

#### Image pyramid multi-scale input

The input of the DDSC-Net is an image pyramid, which can effectively improve the segmentation quality of the network. This method employs the average pooling layer to build an image pyramid, which is then introduced into different layers of the encoder module. The advantages of this are as follow: (1) to avoid a large increase in network parameters; (2) increase the network width of the decoder depth; (3) and reduce the loss of information caused by the deepening of the network.

#### DDSC network

Inspired by U-net [15], a fully convolutional network with a U-shaped structure that using skip connection for feature fusion in each stage, we designed the DDSC network structure based on the U-net structure. See from Fig. 4. The DDSC network consists of an encoder and a decoder connected by skip connection. Specifically, the encoder is employed to extract the high-level semantic features of the input image, and the decoder is adopted to restore the semantic features extracted by the encoder to the resolution of the original image. Skip connection is utilized to fuse multi-scale features between encoder and decoder. Different from the original U-net, in our proposed network, we employ depthwise separable convolution layers to replace most of the standard convolutional layers in the network, which can significantly reduce the amount of computation. Therefore, we design a deeper network to learn more feature information from input data, especially the semantics of the optic cup. In addition,we execute more skip connections between encoder and decoder to enhance the transfer of contextual feature information in our model. The DDSC network is composed of three parts: densely connected depthwise separable convolution blocks, subsampled layers and upsampling layers. A dense depthwise separable convolution (DDSC) block contains five densely connected layers which consist of a batch normalization layer, a rectified linear unit (Relu) activation function, and a depthwise separable convolution layer with kernel size of $3\times 3$. The subsampled layer is a max pooling layer with kernel size of 2 and stride of 2. And the upsampling layer is a $3\times 3$ transposed convolution layer.

For standard convolution, the output feature map *F* for standard convolutional when assuming stride and padding as one is computed as:

$$\begin{aligned} F_{k, l, n}=\sum _{i, j, m} K_{i, j, m, n} \cdot I_{k+i-1, l+j-1, m} \end{aligned}$$

The parameters and computational cost of the standard convolutions are respectively computed as:

$$\begin{aligned} k \times k \times M \times N \end{aligned}$$

and

$$\begin{aligned} k \times k \times M \times N \times H \times W \end{aligned}$$

where *I* is the input feature map or input image, *K* is the convolution kernel size with $k \times k$, *M* is the number of input channel, *N* is the number of output channel, *H* and *W* are the height and width of the input feature map or input image respectively. While depthwise separable convolution is made of depthwise and pointwise convolutions [26]. The output feature map *F* for depthwise separable convolutional is computed as:

$$\begin{aligned} F_{k, l, n}^{\prime }=\sum _{i, j} K_{i, j, m}^{\prime } \cdot I_{k+i-1, l+j-1, m} \end{aligned}$$

And the parameters and computational cost of the depthwise separable convolutions are respectively computed as:

$$\begin{aligned} k \times k \times M + M \times N \end{aligned}$$

and

$$\begin{aligned} k \times k \times M \times H \times W+M \times N \times H \times W \end{aligned}$$

Comparing the parameters of the depthwise separable convolution with the standard convolution can be obtained as follows:

$$\begin{aligned} \frac{k \times k \times M + M \times N }{ k \times k \times M \times N }=\frac{1}{N}+\frac{1}{k^{2}} \end{aligned}$$

It can be seen that the depth separable convolution uses about 8 to 9 times less parameter than the standard convolution. Therefore, we can deepen and widen the network without causing an explosive increase in the number of parameters, and also enable the network to learn more contextual information.

### Post-processing

The output of the network is a map with resolution of $240\times 240$. We used cubic interpolation to restore it to $480\times 480$ and $560 \times 560$. Then adopted morphological operations to smooth the edges. There are four kinds of operation methods of image morphology: erode, dilate, open and close. Based on the prior knowledge that most of the optic disc and cup are elliptical structure, we use the closed operation in the image morphology to fuse the pixel points with fine boundary connection and fill the concave angle of the image, so as to make the boundary of the segmented image smoother slippery. The closed operation can be expressed as follows:

$$\begin{aligned} F=(f \oplus s) \ominus s \end{aligned}$$

where *f* is the image, *s* the Structure element, $\oplus$,$\ominus$represent dilate and erode respectively. In our work, *s* is a $7\times 7$ circular structure element.

### Loss function

In our work, we regard the optic disc and optic cup segmentation as a multi-category segmentation task and use One-Hot encoding to process the data. Let $x \in R^{C \times H \times W}$be the input image, and $y \in \left\{ y_{o}, \ldots , y_{i}\right\} ^{i \times H \times W}$ is the One-Hot representation of the ground truth label, when the pixel belong to category *i*, $y_{i}=1$,otherwise, $y_{i}=0$. We treat the output as 3 categories $i=3$ and the output of our model is a map of $f_{i}(x, v)=y^{\prime } \in \left\{ p_{o}, \ldots , p_{i}\right\} ^{i \times C \times W}$. In our work, we use Multi-class cross-entropy loss function to measure the difference between the output of the model and the ground truth label. The loss function Loss is defined as:

$$\begin{aligned} {Loss}=-\sum _{i=0}^{2} y_{i} \log \left( f_{i}(x, v)\right) \end{aligned}$$

The output map $f_{i}(x, v)$is a probability distribution, and each element$\left\{ p_{o}, \ldots , p_{i}\right\} ^{i \times C \times W}$ represents the probability that the pixel belongs to the $i-th$ category.

## Experiments and results

In this section, we firstly introduce the details of experiment implementation, then state the evaluation metrics. Finally, experimental results are given and discussed.

### Implementation detail

All experiments are implemented in Python with the Pytorch framework on the workstation of Intel i7-8700K, 16G RAM, Nvidia 1080Ti GPU and Ubuntu16.04. The Adam [27] optimizer and back-propagation are employed to train our model. The initial learning rate is set to 1e-4 and decreased by a factor of 10 every 4 epoch. We train 30 epochs with a batch size of 8. Early Stopping was adopted in the training, and the best performing model is taken as the final model. We adopt a cross-validation method to train our model, therefore, the training images are divided into 90$\%$ for training and 10$\%$ for validation after data augmentation. Both training and validation images are resized into the resolution of $240\times 240$ and then fed to the network for training. Figure 5 shows the training of our model on the GS dataset. As we can see from Fig. 5, our model has converged after training for 10,000 iterations. The loss of the training set and the validation set are basically the same, indicating that our model is easy to converge. We have released our codes on Github: https://github.com/iceyanGG/DDSC-NET.

### Evaluation metrics

In our paper, we adopt the Dice coefficients (DC), Jaccard (JAC), Sensitivity (SEN) and Precision (PRE) to evaluate the segmentation performance of the presented method. The criteria are defined as:

$$\begin{aligned} {Dice}= & {} \frac{2 \times N_{t p}}{2 \times N_{t p}+N_{f p}+N_{f n}}\\ {Jaccard}= & {} \frac{N_{t p}}{N_{t p}+N_{f p}+N_{f n}} \\ {Sensitivity}= & {} \frac{N_{t p}}{N_{t p}+N_{f n}} \\ {Precision}= & {} \frac{N_{t p}}{N_{t p}+N_{f p}} \end{aligned}$$

where the $N_{t p}, N_{f p}, N_{f n}$ represent the number of true positive, false positive and false negative pixels, respectively.

### Experimental results

To verify the effectiveness of our algorithm, we perform a lot of comparative experiments. Ablation experiments are firstly conducted to compare the performance with and without image pyramid input, multi-DDSC blocks, and post-processing in the model. Then, we compared the performance of our method with some state-of-the-art deep learning-based methods. Finally, we compared our segmentation results with the results of the REFUGE challenge. Testing images of DRISHTI-GS and REFUGE datasets are employed to evaluate the performance of our model, and the final evaluation scores are the average of all the testing images of the dataset, respectively. The final experimental results of the proposed method are shown in Table 3. On the Drishti-GS dataset, our method obtains DC, JAC, sensitivity and precision of 0.9780, 0.9570, 0.9784 and 0.9778 on OD segmentation results and 0.9123, 0.8442, 0.9220 and 0.9149 on OC segmentation results, respectively. On REFUGE dataset, our method achieves DC, JAC, sensitivity and precision of 0.9601, 0.9239, 0.9814 and 0.9412 on OD segmentation results and 0.8903, 0.8065, 0.9209 and 0.8749 on OC segmentation results, respectively. Figures 6 and 7 show some visual examples of the segmentation in DRISHTI-GS and REFUGE dataset, respectively.

#### Ablation experiment results

In the ablation experiments, 4 comparative experiments were conducted to verify the effectiveness of our proposed method, include DDSC-Net without image pyramid input, simple network with only one DDSC blocks in each layer, DDSC-Net, and DDSC-Net with post-processing. Table 4 summaries the ablation results of the OD and OC segmentation on the Drishti-GS dataset and REFUGE dataset. From Table 4 we can see that both image pyramid input and multi-DDSC blocks can improve the performance of the model, and the proposed post-process method can further improve the segmentation results. Specifically, on the Drishti-GS dataset, when image pyramid is employed as the input of the DDSC-Net, the DC scores of the model are 0.36$\%$ (OD) and 0.47$\%$ (OC) higher than DDSC-Net without image pyramid inputs, and also outperform the simple network which has only one DDSC blocks in each layer by 0.33 $\%$ (OD) and 1.8$\%$ (OC). When post-processing is utilized, the segmentation performance is further improved. For example, on the REFUGE dataset, the OD and OC segmentation results on DC are increased by 0.09$\%$ (OD) and 0.09$\%$ (OC), respectively.

Some visual examples are shown in Fig. 7. We selected 8 representative images among the 400 REFUGE test images to show the segmentation results. The first two rows are the cropped fundus images and ground truth, and the remaining 4 rows are the experimental results of 4 comparative experiments. As can be seen from Fig. 7, using image pyramid input to replace standard input can effectively improve model performance, and using multiple DDSC blocks can also improve model performance.

#### Compared with deep learning-based methods

In order to verify that our method is better than other deep learning-based methods, we compare our proposed method with the state-of-the-art approaches, such as pOSAL framework [28], GL-Net [29], M-Net [16], Stack-U-Net [30], WGAN [31], two-stage Mask R-CNN [32], multi-modal self-supervised pre-training network [33], Yu et al. [34] and Sevastopolsky [10]. Additionally, we compare with the Fully convolutional network U-Net [15]. The segmentation results of these deep learning-based methods on the testing set of Drishti-GS and REFUGE dataset are shown in Table 5 and 6, respectively.

Since our network structure is modified based on the U-Net architecture, we firstly compare our model with other methods that also use the U-shaped network. Firstly, we compare with the original U-net on the Drishti-GS dataset. From Table 5, we can see that the proposed method achieves 2.8$\%$ and 9.23$\%$ higher than the original U-net in the Dice coefficients of the optic disc and the optic cup, respectively. When compared with the same methods modified based on the U-net structure [10, 16, 30, 34], our method outperform the best performance method proposed by Yu et al. [34], which is 0.42$\%$ higher in OD, and 2.46$\%$ in the more difficult OC segmentation task. On the REFUGE dataset, our method is also better than that of M-net [16]. From Table 6 we can see that our method is 1.65$\%$ higher in the optic disc and 5.88$\%$ in the optic cup compared to M-Net.

GAN-based methods and other deep learning networks also achieved satisfactory segmentation results in OD and OC segmentation task. Therefore, we then compared the use of generative adversarial ideas, WGAN [28], GL-Net [29] and pOSAL framework [31] and other deep learning methods [32, 33]. From Table 5 and 6, we can see that our proposed method still performs better than these methods. Particularly in the optic disc and cup segmentation results on the Drishti-GS dataset, our model outperforms the state-of-the-art method GL-Net by 0.7$\%$ and 0.73$\%$ in term of dice coefficients, respectively. These comparative results demonstrate that our proposed method can effectively improve the segmentation accuracy of the OC and obtain competitive results in the segmentation of the OD.

#### Compared with REFUGE challenge

We also compared our segmentation results with the results of the REFUGE challenge. The segmentation results of the 12 participating teams are shown in Table 7. It is obvious that in terms of DC metric our method achieves the best segmentation result in the segmentation of the cup, which is 0.66$\%$. higher than the best result of 0.8837. In the segmentation of the optic disc, we achieved the second-best segmentation result, showing strong competitiveness.

From the above, it can be concluded that our method can effectively segment the optic disc and the optic cup, especially in the segmentation task of OC, and achieved the start-of-the-art segmentation performance on the Drishti-GS and REFUGE dataset.

## Discussion

In this paper, a densely connected depthwise separable convolution deep network for joint OD and OC segmentation method is proposed. As experimental results and comparative experiments above, we can draw a conclusion that Multi-scale image pyramid input and densely connected deep separable network can effectively perform OD and OC segmentation. The correct segmentation of optic disc and optic cup is essential for calculating CDR, which helps clinicians diagnose glaucoma more effectively. Because OC is located in OD and there is no obvious boundary like OD, OC segmentation has always been a more challenging task in the segmentation task of OD and OC. In prior segmentation work, the proposed method could not obtain satisfactory OC segmentation results, whereas the network we designed can effectively improve the OC segmentation results. According to our experimental results, using DDSC blocks to deepen the network and enhance the fusion of contextual semantic information through dense connections and skip connections can effectively improve the segmentation effect. Compared with the current optimal OD and OC segmentation network, our method obtains the state-of-the-art segmentation results both in two public datasets. Our DDSC module is formed by densely connected deep separable convolution, which has 8 to 9 times fewer parameters than standard convolution. Therefore, the advantage of using the DDSC module is that it can deepen the network depth and learn deeper semantic information for feature fusion without increasing the network parameters.

Although our method has achieved the best OD and OC segmentation results on the testing images of two public datasets. However, our proposed method still has limitations. The limitations of our research are that for fundus images used by different equipment and institutions, the model parameters we trained cannot perform stable segmentation on fundus images of these different shooting devices. To use our method to perform segmentation on fundus images of a new dataset, the network parameters need to be retrained. Improving the generalization of the model and stably segmenting fundus images from different data sets are also our future research directions.

## Conclusion

The research on joint OD and OC segmentation is an important part of the field of medical image processing and of great importance to computer-aided glaucoma technologies. In this paper, we aim at exploring a novel automatic OD and OC segmentation method based on deep learning techniques to improve the performance of CAD system for diagnosing glaucoma, which can be applied to assist clinicians in the diagnosis of glaucoma and population screening. The proposed network employs a dense connection of deep separable convolution network as the backbone network and adds a multi-scale image pyramid at the input end to widen the network. Finally, image morphology is employed to post-process the segmentation results. To verify the effectiveness of our proposed DDSC-Net, we conduct ablation experiments and compare our method with previous methods on the DRISHTI-GS and REFUGE dataset. The experimental results show that our model outperforms the state-of-the-art method on the DRISHTI-GS and REFUGE dataset, which show better potential for improving the accuracy of the CAD system in diagnosing glaucoma. In future research work, we will try to apply our method to other medical segmentation tasks, such as retinal vessel segmentation, liver lesion segmentation, etc. Besides, we will pay more attention to solving the problem of domain shift between different datasets, so as to improve the generalization performance of the proposed method.

## Data Availability

The datasets are publicly available at the follow links: (1) DRISHTI-GS dataset: http://cvit.iiit.ac.in/projects/mip/drishti-gs/mip-dataset2/Home.php; (2) REFUGE dataset: https://refuge.grand-challenge.org.

## Abbreviations

IOP: Intraocular pressure assessment
ONH: Optic nerve head
CDR: Cup to disc ratio
OD: Optic disc
OC: Optic cup
CAD: Computer aided diagnosis
CNN: Convolution neural network
ROI: Region of interest
CHT: Circular Hough transform
DC: Dice coefficients
JAC: Jaccard
SEN: Sensitivity
PRE: Precision

## References

[1] Tham YC, Li X, Wong TY, Quigley HA, Aung T, Cheng CY (2014). Global prevalence of glaucoma and projections of glaucoma burden through 2040: a systematic review and meta-analysis. Ophthalmology.

[2] Quigley HA, Broman AT (2006). The number of people with glaucoma worldwide in 2010 and 2020. Br J Ophthalmol.

[3] Michelson G, Wärntges S, Hornegger J, Lausen B (2008). The papilla as screening parameter for early diagnosis of glaucoma. Dtsch Rzteblatt Int.

[4] Aquino A, Gegundez-Arias ME, Marin D (2010). Detecting the optic disc boundary in digital fundus images using morphological, edge detection, and feature extraction techniques. IEEE Trans Med Imaging.

[5] Dashtbozorg B, MendonçA AM, Campilho A (2015). Optic disc segmentation using the sliding band filter. Comput Biol Med.

[6] Chakravarty A, Sivaswamy J (2017). Joint optic disc and cup boundary extraction from monocular fundus images. Comput Methods Programs Biomed.

[7] Zheng Y, Stambolian D, O’Brien J, Gee JC Optic disc and cup segmentation from color fundus photograph using graph cut with priors. In: International conference on medical image computing and computer-assisted intervention. 2013. 10.1007/978-3-642-40763-5_10 PMC4165089 24579126

[8] Zilly J, Buhmann JM, Mahapatra D (2017). Glaucoma detection using entropy sampling and ensemble learning for automatic optic cup and disc segmentation. Comput Med Imaging Graph.

[9] Maninis K-K, Pont-Tuset J, Arbeláez P, Van Gool L. Deep retinal image understanding. In: International conference on medical image computing and computer-assisted intervention. Springer; 2016. p. 140–8.

[10] Sevastopolsky A (2017). Optic disc and cup segmentation methods for glaucoma detection with modification of u-net convolutional neural network. Pattern Recogn Image Anal.

[11] Shankaranarayana SM, Ram K, Mitra K, Sivaprakasam M. Joint optic disc and cup segmentation using fully convolutional and adversarial networks. In: Fetal, infant and ophthalmic medical image analysis. Cham: Springer; 2017. p.168–76.

[12] Krizhevsky A, Sutskever I, Hinton GE. Imagenet classification with deep convolutional neural networks. In: Advances in neural information processing systems. 2012. p. 1097–1105.

[13] Long J, Shelhamer E, Darrell T. Fully convolutional networks for semantic segmentation. In: Proceedings of the IEEE conference on computer vision and pattern recognition. 2015. p. 3431–3440. 10.1109/TPAMI.2016.2572683 27244717

[14] Simonyan K, Zisserman A. Very deep convolutional networks for large-scale image recognition. 2014. arXiv:1409.1556.

[15] Ronneberger O, Fischer P, Brox T. U-net: convolutional networks for biomedical image segmentation. In: International conference on medical image computing and computer-assisted intervention. Springer; 2015. p. 234–241.

[16] Fu H, Cheng J, Xu Y, Wong DWK, Liu J, Cao X (2018). Joint optic disc and cup segmentation based on multi-label deep network and polar transformation. IEEE Trans Med Imaging.

[17] Sivaswamy J, Krishnadas S, Joshi GD, Jain M, Tabish AUS Drishti-gs: Retinal image dataset for optic nerve head (onh) segmentation. In: 2014 IEEE 11th international symposium on biomedical imaging (ISBI). IEEE; 2014. p. 53–56. 10.1109/ISBI.2014.6867807.

[18] Orlando JI, Fu H, Breda JB, van Keer K, Bathula DR, Diaz-Pinto A, Fang R, Heng P-A, Kim J, Lee J (2020). Refuge challenge: a unified framework for evaluating automated methods for glaucoma assessment from fundus photographs. Med Image Anal.

[19] Abdullah M, Fraz MM, Barman SA (2016). Localization and segmentation of optic disc in retinal images using circular hough transform and grow-cut algorithm. PeerJ.

[20] Hough P. Method and means for recognizing complex pattern. 1962.

[21] Duda RO, Hart PE (1972). Use of the hough transformation to detect lines and curves in pictures. Commun. ACM.

[22] Liu W, Anguelov D, Erhan D, Szegedy C, Reed S, Fu C-Y, Berg AC. Ssd: single shot multibox detector. In: European conference on computer vision. Springer; 2016. pp. 21–37.

[23] Redmon J, Farhadi A. YOLOv3: an incremental improvement. 2018. arXiv:1804.02767.

[24] Bochkovskiy A, Wang C-Y, Liao H-YM. YOLOv4: optimal speed and accuracy of object detection. 2020. arXiv:2004.10934.

[25] Adelson EH, Anderson CH, Bergen JR, Burt PJ, Ogden JM (1984). Pyramid methods in image processing. RCA Eng.

[26] Howard AG, Zhu M, Chen B, Kalenichenko D, Wang W, Weyand T, Andreetto M, Adam H. MobileNets: efficient convolutional neural networks for mobile vision applications (2017). arXiv:1704.04861.

[27] Kingma DP, Ba J. Adam: a method for stochastic optimization. 2014. arXiv:1412.6980.

[28] Wang S, Yu L, Yang X, Fu C-W, Heng P-A (2019). Patch-based output space adversarial learning for joint optic disc and cup segmentation. IEEE Trans Med Imaging.

[29] Jiang Y, Tan N, Peng T (2019). Optic disc and cup segmentation based on deep convolutional generative adversarial networks. IEEE Access.

[30] Sevastopolsky A, Drapak S, Kiselev K, Snyder BM, Keenan JD, Georgievskaya A. Stack-u-net: refinement network for improved optic disc and cup image segmentation. In: Medical Imaging 2019: Image Processing, vol. 10949. International Society for Optics and Photonics; 2019. p. 1094928. 10.1117/12.2511572.

[31] Kadambi S, Wang Z, Xing E (2020). Wgan domain adaptation for the joint optic disc-and-cup segmentation in fundus images. Int J Comput Assist Radiol Surg.

[32] Almubarak H, Bazi Y, Alajlan N (2020). Two-stage mask-rcnn approach for detecting and segmenting the optic nerve head, optic disc, and optic cup in fundus images. Appl Sci.

[33] Hervella Á S, Ramos L, Rouco J, Novo J, Ortega M. Multi-modal self-supervised pre-training for joint optic disc and cup segmentation in eye fundus images. In: ICASSP 2020-2020 IEEE international conference on acoustics, speech and signal processing (ICASSP). IEEE; 2020. p. 961–965. 10.1109/ICASSP40776.2020.9053551.

[34] Yu S, Xiao D, Frost S, Kanagasingam Y (2019). Robust optic disc and cup segmentation with deep learning for glaucoma detection. Comput Med Imaging Graph.

